# Evaluation of the prevention effect of high-quality nursing quality control in disinfection supply center on nosocomial infection

**DOI:** 10.1097/MD.0000000000035459

**Published:** 2024-01-12

**Authors:** Ping Yu, Rong Yang, Changfei Cen

**Affiliations:** a Central Sterile Supply Department, Renhe Hospital Affiliated to China Three Gorges University, Yichang, Hubei, China; b Department of Nursing, Renhe Hospital Affiliated to China Three Gorges University, Yichang, Hubei, China; c Department of Critical Care Medicine, Renhe Hospital Affiliated to China Three Gorges University, Yichang, Hubei, China.

**Keywords:** application effect, disinfection supply center, high-quality nursing quality control

## Abstract

To explore the application effect of high-quality nursing quality control in disinfection supply center. The control group consisted of 1850 medical devices managed using the conventional quality control mode from January 2021 to December 2021, while the observation group consisted of 1900 medical devices managed using the high-quality nursing quality control mode from January 2022 to December 2022. The qualified rates of equipment cleaning, sterilization, and packaging were analyzed in both the observation and control groups. The occurrence of nosocomial infections in 2021 and 2022 were compared, and the changes in the Beck-Srivaatava stress scale index (BSSI) and Symptom Checklist-90 scores of the staff before and after implementing the high-quality nursing quality control mode were analyzed. The qualified rate of equipment cleaning, sterilization, and packaging in the observation group were 99.08%, 99.73%, and 99.78%, respectively, which were significantly higher than those in the control group (*P* < .05). The incidence of nosocomial infections in interventional and surgical cases in 2022 was 0.79%, which was significantly lower than that in 2021 (*P* < .05). The BSSI score of female staff was (68.76 ± 7.81) points, which was higher than that of male staff (*P* < .05). After the implementation of the high-quality nursing quality control mode, the BSSI score of the staff was (47.76 ± 9.12) points, which was significantly lower than that before implementation (*P* < .05). After the implementation of the high-quality nursing quality control mode, the staff’s Symptom Checklist-90 scores for somatization, compulsion, interpersonal sensitivity, depression, hostility, and paranoia were (1.28 ± 0.29), (1.53 ± 0.24), (1.50 ± 0.21), (1.46 ± 0.32), (1.44 ± 0.26), and (1.38 ± 0.30) points, respectively, showing a decrease compared to before implementation (*P* < .05). The high-quality nursing quality control mode has great application value in the disinfection supply center. It can effectively improve the qualified rates of equipment cleaning, sterilization, and packaging, prevent nosocomial infections and improve the working pressure and psychological health of staff.

## 1. Introduction

The disinfection supply center is primarily responsible for cleaning, disinfecting, and sterilizing medical supplies or equipment, ensuring that they achieve a sterile state, and providing sterile medical equipment to various departments of the hospital.^[1–3]^ In addition, the disinfection supply center is also the main department for handling contaminated medical materials, so improving the management quality of the disinfection supply center is of great significance for ensuring the safety and reliability of medical supplies. High-quality nursing quality control refers to placing more emphasis on the responsibilities of nursing staff on the basis of routine nursing, ensuring that nursing services are more professional and of higher quality. Its purpose is to improve the quality and overall level of work in the disinfection supply center, provide clinical safety of medical supplies, reduce the incidence of hospital infections, prevent adverse events, and ensure a safe medical environment, while also improving the level of medical care.^[4–6]^ Based on this, the aim of this study was to explore the application effect of high-quality nursing quality control in the disinfection supply center. The results are reported below.

## 2. Data and methods

### 2.1. General data

This study was approved by the Ethics Committee of Renhe Hospital affiliated to China 3 Gorges University. The control group consisted of 1850 medical devices managed using routine quality control mode from January 2021 to December 2021, while the observation group consisted of 1900 medical devices managed using high-quality nursing quality control mode from January 2022 to December 2022. The composition of medical devices in the observation group and control group was compared (*P* > .05), see Table 1. Simultaneously, 30 disinfection supply center staff members were selected for a questionnaire survey during the same period, including 10 males and 20 females, with ages ranging from 21 to 44 years and a mean age of (32.52 ± 4.15) years. In terms of educational background, there were 11 staff members with a secondary vocational education, 12 staff members with a junior college education, and 7 staff members with a bachelor’s degree or higher. Inclusion criteria: Working years in the disinfection supply center ≥ 1 year; All have relevant work qualification certificates; Voluntary participation in this study and signed informed consent. Exclusion criteria: Those with serious diseases such as malignant tumors; Negative life events occurred in the past year; Patients with a history of mental illness.

**Table 1:** Comparison of medical instruments composition between observation group and control group.

Group	Number of instruments	Type of instruments				
		Hemostatic forceps, scissors, tweezers (pieces)	Container class (piece)	Cavity mirror (piece)	Laryngoscope (piece)	Other (piece)
Observation group	1850	672 (36.32)	551 (29.78)	334 (18.05)	190 (10.27)	103 (5.57)
Control group	1900	655 (34.47)	564 (29.68)	328 (17.26)	224 (11.79)	129 (6.79)
c^2^		5.464				
*P* value		.243				

### 2.2. Intervention methods

The control group was managed using a routine quality control mode: disinfection work was carried out according to the hospital’s work system and procedures.

The observation group was managed using a high-quality nursing quality control mode: An excellent nursing quality control team was established. The team held meetings to discuss and develop a detailed work plan, and conducted regular training and learning activities to impart the latest knowledge and skills in equipment management, disinfection, and infection control to nursing staff. The team also organized nursing staff to participate in lectures and discussions, inviting experts, scholars, or senior figures in the industry to provide professional explanations and exchanges on equipment management, infection prevention, and other aspects. After the meetings, team members provided detailed explanations and training to nursing staff on the principles and methods of disinfection and sterilization. They also strengthened guidance and supervision during practical operations, and organized inspections and evaluations of the effectiveness of disinfection and sterilization to ensure the quality and safety of disinfection and sterilization work; Through conducting research, an understanding of the actual situation in the hospital and center was gained, the problems and shortcomings of the existing system were analyzed, and based on the research results and actual situation, a new set of rules and regulations was formulated. These rules and regulations clearly defined the responsibilities, work processes, and operational standards of the disinfection supply center, ensuring that the staff had a clinical basis during the operation process. After the new set of rules and regulations was formulated, relevant experts and leaders were invited to review and modify them, ensuring the rationality and operability of the regulations. After the new set of rules and regulations were established, relevant training and publicity was provided to the staff; Performance assessment indicators and reward standards were set up based on work performance, work quality and work attitude. Excellent employees with outstanding performance were rewarded accordingly. Regular performance assessments were conducted, and based on the results, corresponding rewards and punishments were given. The assessment covered multiple aspects, including work quality, work efficiency, work attitude, and team cooperation. A punishment system was established to punish behaviors such as noncompliance with rules and regulations, work errors, and other forms of misconduct. Punishment methods could include verbal warnings, written warnings, fines, salary reductions, demotions, etc, based on the circumstances, with appropriate punishments given; Complete records and monitoring were conducted for all instrument cleaning procedures carried out in clinical areas to trace the entire process of each instrument from cleaning to use, including cleaning, disinfection, drying, packaging, storage, and other links. This ensured that each step adhered to the requirements of the rules and regulations, and improvements were made to address any issues that arose during actual work. A traceability mechanism was established to investigate instruments involved in past infection events, identify the source of infection, investigate the causes, and take timely measures to prevent similar incidents from reoccurring. Regular environmental cleaning was carried out, and standardized management of cleaning and disinfection procedures implemented in the department to ensure that all cleaning and disinfection work complied with the requirements of the rules and regulations; A professional psychological questionnaire was used to conduct a comprehensive psychological evaluation of the nurses in the disinfection supply center, including their emotional state, stress levels, and mental health. Regular counseling sessions were conducted for the nurses by psychological experts to monitor their life habits, economic conditions, cultural level, and acceptance level. The nurses psychological reactions and needs were taken into consideration, and timely psychological guidance and assistance were provided to them.

### 2.3. Assessment methods

The Beck-Srivaatava stress scale index (BSSI)^[7]^ was used to assess the stress levels of the staff, with a total score of 138 points where a higher score indicates higher stress levels. Scores below 35 indicate mild stress, scores between 36 to 70 indicate moderate stress, and scores between 71 to 138 indicate severe stress; The symptom checklist-90 (SCL-90)^[8]^ was used to evaluate the mental health of the staff, which includes 9 symptom factors, such as somatization, obsession-compulsion, interpersonal sensitivity, depression, anxiety, hostility, phobic anxiety, paranoid ideation, and psychoticism. Each factor is rated on a scale from 1 to 5, with a higher score indicating a poorer mental health condition.

### 2.4. Statistical processing

SPSS 22.0 software was used. The measurement data were expressed as (X̄± s), and the difference between groups was analyzed by *t* test. The count data were expressed as n (%), and the difference between groups was analyzed by χ^2^ test.

## 3. Results

### 3.1. Comparison of qualified cleaning of instruments between observation group and control group

The observation group showed significantly higher rates of qualified instrument cleaning, sterilization, and packaging than the control group (*P* < .05). See Table 2

**Table 2:** Comparison of qualified cleaning of instruments between observation group and control group.

Group	Number of instruments	Rates of qualified instrument cleaning (%)	Rates of qualified instrument sterilization (%)	Rates of qualified instrument packaging (%)
Observation group	1850	1833 (99.08)	1845 (99.73)	1846 (99.78)
Control group	1900	1765 (92.89)	1770 (93.16)	1768 (93.05)
c^2^		92.24	116.651	121.509
*P* value		.000	.000	.000

### 3.2. Comparison of the incidence of nosocomial infection in different time periods

The incidence of nosocomial infections in interventional and surgical cases in 2022 was significantly lower than that in 2021 (*P* < .05). See Table 3

**Table 3:** Comparison of the incidence of nosocomial infection in different time periods.

Year	Intervention and operation person-times	Incidence of nosocomial infections (%)	c^2^	*P* value
2021	3933	56 (1.42)	7.667	.006
2022	4204	33 (0.79)		

### 3.3. Comparison of BSSI scores of different staff before the implementation of high-quality nursing treatment control mode

Female staff had significantly higher BSSI scores than male staff (*P* < .05); BSSI scores were compared among staff of different ages and educational backgrounds (*P* > .05). See Table 4

**Table 4:** Comparison of BSSI scores of different staff before the implementation of high-quality nursing treatment control mode.

Group	Number of cases	BSSI score (score)	t	*P* value
Gender				
Male	10	62.28 ± 8.27	−2.102	.045
Female	20	68.76 ± 7.81		
Age				
<30 yr old	12	64.40 ± 8.05	−1.21	.236
≥30 yr old	18	68.07 ± 8.19		
Education background				
College or higher	19	66.12 ± 8.18	−0.407	.687
Secondary technical school	11	67.43 ± 9.04		

BSSI = Beck-Srivaatava stress scale index.

### 3.4. Comparison of BSSI scores of staff before and after the implementation of high-quality nursing quality control mode

After the implementation of the high-quality nursing quality control mode, the BSSI scores of the staff were markedly lower than before the implementation (*P* < .05). See Table 5

**Table 5:** Comparison of BSSI scores of staff before and after the implementation of high-quality nursing quality control mode.

Time	Number of cases	BSSI score (score)	t	*P* value
Before implementation	30	66.60 ± 9.94	7.649	.000
After implementation	30	47.76 ± 9.12		

BSSI = Beck-Srivaatava stress scale index.

### 3.5. Comparison of SCL-90 scores of staff before and after the implementation of high-quality nursing quality control mode

After the implementation of the high-quality nursing quality control mode, the staff’s SCL-90 scores for somatization, compulsion, interpersonal relation, depression, hostility, and paranoid ideation were lower than before the implementation (*P* < .05). See Table 6

**Table 6:** Comparison of SCL-90 scores of staff before and after the implementation of high-quality nursing quality control mode.

SCL-90 item	Before implementation (n = 30)	After implementation (n = 30)	t	*P* value
Somatization (score)	1.60 ± 0.30	1.28 ± 0.29	4.201	.000
Compulsion (score)	1.80 ± 0.27	1.53 ± 0.24	4.094	.000
Interpersonal relation (score)	1.89 ± 0.25	1.50 ± 0.21	6.543	.000
Depression (score)	1.92 ± 0.30	1.46 ± 0.32	5.744	.000
Anxiety (score)	1.62 ± 0.32	1.60 ± 0.30	0.250	.804
Hostility (score)	1.72 ± 0.29	1.44 ± 0.26	3.938	.000
Horror (score)	1.44 ± 0.30	1.42 ± 0.28	0.267	.790
Paranoid ideation (score)	1.69 ± 0.27	1.38 ± 0.30	4.207	.000
Psychotic diseases (score)	1.40 ± 0.22	1.35 ± 0.21	0.900	.372

SCL-90 = symptom checklist-90.

## 4. Discussion

The disinfection supply center is an important part of medical institutions. Its responsibility is to ensure the safety and reliability of medical devices, provide high-quality disinfection services, and provide strong support for the hospital’s infection prevention work.^[9–11]^ Nosocomial infections are an important factor affecting the quality of medical care, and their harmfulness and scope of influence cannot be underestimated. The importance of nosocomial infection prevention in hospitals is increasingly being recognized. Therefore, staff need to strictly follow the procedures when organizing and disinfecting medical supplies to ensure the safety and reliability of medical supplies. However, due to the large amount of medical equipment that needs to be processed every day, and the time urgency and heavy workload that staff face, this may cause stress, and negative emotions, which may lead to a decrease in work quality.^[12–15]^ The implementation of high-quality nursing quality control aims to improve the quality of service to provide strong support for hospital infection prevention by standardizing operational procedures, strengthening training and learning, establishing service evaluation mechanisms, among other measures. This approach enhances the qualifications of nursing staff, cultivates their professional skills, improves their service awareness and service quality, and ultimately improves the supply and management of medical equipment and disinfectants. In addition, high-quality nursing quality control places more emphasis on the work environment and mental health of nurses, providing necessary support and resources to help them effectively cope with work-related stress and emotional distress. This ultimately helps to improve work efficiency and quality.^[16–18]^

The results of this study show that the observation group’s rates of qualified instrument cleaning, sterilization, and packaging were significantly higher than those of the control group (*P* < .05). This suggests that high-quality nursing quality control has a significant impact on the quality of instrument cleaning, sterilization, and packaging. The improvement in quality can be attributed to the observation group’s detailed work plans, group discussions, and training programs that ensure that all nursing staff are up-to-date with the latest knowledge and skills in instrument management, disinfection, and infection control. The regular training and learning activities can further enhance the professional level of nursing staff, strengthen their theoretical foundation and operational skills in disinfection and sterilization work.^[19,20]^ Inviting expert scholars or seasoned professionals for lectures and exchanges can improve nursing staff’s understanding and cognition of disinfection and sterilization work, thereby improving their accuracy and consistency in practical operations.^[21–23]^ Post-conference lectures and training can ensure that nursing staff master the correct operating procedures. In addition, spot-checks and evaluations can help detect problems promptly and corrective actions can be taken. Moreover, setting performance evaluation indicators and reward criteria can motivate staff to work harder and be more focused. Providing corresponding rewards and recognition to outstanding employees can encourage them to continuously improve their work level and enhance their motivation and sense of responsibility towards work. The establishment of a punishment system can also compel staff to strictly adhere to operational norms, reducing work errors and preventing the occurrence of misconduct.^[24–26]^

The results of this study also showed that the incidence of hospital-acquired infections in 2022 was significantly lower than that in 2021 (*P* < .05). This suggests that high-quality nursing quality can effectively reduce the incidence of hospital-acquired infections. The reason for this improvement is likely due to conducting surveys to understand the actual situation of the hospital and disinfection supply center, which can help identify the direction and focus of improvements. This ensures that staff have clinical basis and guidelines during the operational process, thereby reducing the arbitrariness and uncertainty of operations. The establishment of a complete quality management system through recording and monitoring can improve the traceability and controllability of instrument cleaning work. Tracing the instruments that caused infection events, and identifying the source of infection can increase staff’s awareness of the importance of instrument cleaning work, promoting implementation of responsibilities and problem-solving. Furthermore, regular cleaning and disinfection can effectively reduce the presence of pollutants and pathogens in the environment, reducing the risk of hospital-acquired infections.^[27–30]^

This study used the BSSI scale to assess the stress levels of staff, providing a basis for corresponding intervention measures. The results showed that women staff had higher BSSI scores than men, indicating that women are more susceptible to stress in the workplace. Therefore, psychological interventions should be inclined towards women. This study also showed that after implementation of the nursing quality control model, staff BSSI scores and SCL-90 scores for some items were significantly lower than before implementation (*P* < .05). This is because the observation group organized personalized counseling and guidance from psychological experts for the nurses based on the evaluation results and the individual situation. This helps nurses recognize and cope with their psychological distress and stressors, and provides relevant psychological support and coping strategies to promote psychological health and emotional adjustment. By asking about nurses’ living habits, economic conditions, educational background, and acceptance level, etc, more comprehensive understanding of their living and working environment can be obtained. This is helpful in providing more tailored psychological support and assistance to nurses based on their actual needs, thus improving their psychological state and mental health.^[31–35]^

## 5. Conclusion

High-quality nursing quality control has great application value in the disinfection supply center, which can effectively improve the pass rate of instrument cleaning, sterilization, and packaging, prevent hospital-acquired infections, and improve staff’s work pressure and psychological health.

## Author contributions

**Conceptualization:** Ping Yu.

**Data curation:** Ping Yu.

**Formal analysis:** Rong Yang.

**Investigation:** Rong Yang.

**Supervision:** Changfei Cen.

**Visualization:** Changfei Cen.

**Writing – original draft:** Ping Yu.

**Writing – review & editing:** Ping Yu, Changfei Cen.

## References

[1] Ying J Wang H Ye H. The supervision and management mode of disinfection supply center improves the standardization of sterile goods management in clinical departments. Comput Math Methods Med. 2022;2022:6916212. 35265173 10.1155/2022/6916212 PMC8901292

[2] Wang X Liu XR Li KX. Effects of ferulic acid on regulating the neurovascular unit: implications for ischemic stroke treatment. World J Tradit Chin Med. 2022;8:210–7.

[3] Puig-Asensio M Marra AR Childs CA. Effectiveness of chlorhexidine dressings to prevent catheter-related bloodstream infections. Does one size fit all? A systematic literature review and meta-analysis. Infect Control Hosp Epidemiol. 2020;41:1388–95. 32935659 10.1017/ice.2020.356

[4] Marya NB Muthusamy RV. Methods for endoscope reprocessing. Gastrointest Endosc Clin N Am. 2020;30:665–75. 32891224 10.1016/j.giec.2020.06.002

[5] Yang L Xun Q Xu J. Application of the defect management improvement mode under joint commission international standard to improve the instrument cleaning and disinfection effect and management quality in the central sterile supply department: a randomized trial. Ann Transl Med. 2022;10:137. 35284550 10.21037/atm-21-6610 PMC8904978

[6] Wei Y Xu M Wang W. Effect analysis of Plan-do-check-act cycle method applied in nursing management of disinfection supply room. Panminerva Med. 2022;64:127–9. 32550626 10.23736/S0031-0808.20.03969-5

[7] Abdel Hamid AAL Nasreldin M Gohar SM. Sexual and religious obsessions in relation to suicidal ideation in bipolar disorder. Suicide Life Threat Behav. 2019;49:1552–9. 30729568 10.1111/sltb.12540

[8] Dang W Xu Y Ji J. Study of the SCL-90 scale and changes in the Chinese norms. Front Psychiatry. 2021;11:524395. 33584353 10.3389/fpsyt.2020.524395 PMC7873442

[9] Jing W Mu Y Cai Y. Central sterile supply department (CSSD) management quality sensitive index constructed by management mode under the guidance of key point control theory and its effect on CSSD management quality: a retrospective study. Ann Palliat Med. 2022;11:2050–60. 35817740 10.21037/apm-22-594

[10] Alfred M Catchpole K Huffer E. Work systems analysis of sterile processing: decontamination. BMJ Qual Saf. 2020;29:320–8. 10.1136/bmjqs-2019-009422 PMC7752140 31723018

[11] Yiek WK Coenen O Nillesen M. Outbreaks of healthcare-associated infections linked to water-containing hospital equipment: a literature review. Antimicrob Resist Infect Control. 2021;10:77. 33971944 10.1186/s13756-021-00935-6 PMC8108015

[12] Wu M Wang Q Cheng Z. Cleaning quality control management of medical equipment in hospital disinfection supply room based on smart medicine. Comput Intell Neurosci. 2022;2022:4380735. 36035836 10.1155/2022/4380735 PMC9410962

[13] Boom FA Ris JM Veenbaas T. Reducing the risk of non-sterility of aseptic handling in hospital pharmacies, part B: risk control. Eur J Hosp Pharm. 2021;28:325–30. 32385068 10.1136/ejhpharm-2019-002179 PMC8552189

[14] Shaban RZ Sotomayor-Castillo C Nahidi S. Global burden, point sources, and outbreak management of healthcare-associated Burkholderia cepacia infections: an integrative review. Infect Control Hosp Epidemiol. 2020;41:777–83. 32441235 10.1017/ice.2020.184

[15] Tršan M Vehovc M Seme K. Evaluation of ATP bioluminescence for monitoring surface hygiene in a hospital pharmacy cleanroom. J Microbiol Methods. 2020;168:105785. 31770539 10.1016/j.mimet.2019.105785

[16] Latha T Bhat AK Hande HM. Effectiveness of extended infection control measures on methicillin-resistant staphylococcus aureus infection among orthopaedic patients. Indian J Orthop. 2022;56:1804–12. 36187590 10.1007/s43465-022-00713-5 PMC9485330

[17] Han Z Pappas E Simmons A. Environmental cleaning and disinfection of hospital rooms: a nationwide survey. Am J Infect Control. 2021;49:34–9. 32798634 10.1016/j.ajic.2020.08.008

[18] Rutala WA Weber DJ. Disinfection and sterilization in health care facilities: an overview and current issues. Infect Dis Clin North Am. 2021;35:575–607. 34362535 10.1016/j.idc.2021.04.004

[19] Crowley P Chatterjee P Coppin JD. Effect of a “feedback prompt” from a disinfection tracking system on portable medical equipment disinfection. Am J Infect Control. 2022;50:1322–6. 35081426 10.1016/j.ajic.2022.01.004 PMC9307688

[20] Banti CN Hadjikakou SK. Antimicrobial materials with medical applications. Int J Mol Sci. 2022;23:1890. 35163811 10.3390/ijms23031890 PMC8837163

[21] Martel JA Chatterjee P Coppin JD. Capturing portable medical equipment disinfection data via an automated novel disinfection tracking system. Am J Infect Control. 2021;49:1287–91. 34565497 10.1016/j.ajic.2021.05.008 PMC8480426

[22] Herraiz-Carboné M Cotillas S Lacasa E. Enhancement of UV disinfection of urine matrixes by electrochemical oxidation. J Hazard Mater. 2021;410:124548. 33246823 10.1016/j.jhazmat.2020.124548

[23] Papp S Kimmerl K Gatz J. Evaluation of sporicidal disinfectants for the disinfection of personal protective equipment during biological hazards. Health Secur. 2020;18:36–48. 32078425 10.1089/hs.2019.0128 PMC7047094

[24] Amodio E Kuster SP Garzoni C. Disinfecting noncritical medical equipment-Effectiveness of hydrogen peroxide dry mist as an adjunctive method. Am J Infect Control. 2020;48:897–902. 32464292 10.1016/j.ajic.2020.05.016

[25] Hu L Wei Y Tu X. [Analysis and research on intelligent management and control system of reusable medical devices]. Zhongguo Yi Liao Qi Xie Za Zhi. 2023;47:154–7. 37096468 10.3969/j.issn.1671-7104.2023.02.008

[26] Sweet W Snyder D Raymond M. Design and implementation of the infection prevention program into risk management: managing high level disinfection and sterilization in the outpatient setting. J Healthc Risk Manag. 2020;40:44–9. 10.1002/jhrm.21407 32367590

[27] Wistrand C Nilsson U Sundqvist AS. Patient experience of preheated and room temperature skin disinfection prior to cardiac device implantation: a randomised controlled trial. Eur J Cardiovasc Nurs. 2020;19:529–36. 32028795 10.1177/1474515119900062

[28] Napolitani M Bezzini D Moirano F. Methods of disinfecting stethoscopes: systematic review. Int J Environ Res Public Health. 2020;17:1856. 32182989 10.3390/ijerph17061856 PMC7143198

[29] Gao B Ge R Zhang D. [Design and implementation of quality and safety traceability system for reusable medical devices disinfection based on RFID technology]. Zhongguo Yi Liao Qi Xie Za Zhi. 2021;45:167–71. 33825376 10.3969/j.issn.1671-7104.2021.02.010

[30] Hetzel-Riggin MD Swords BA Tuang HL. Work engagement and resiliency impact the relationship between nursing stress and burnout. Psychol Rep. 2020;123:1835–53. 31510876 10.1177/0033294119876076

[31] Kunzler AM Helmreich I Chmitorz A. Psychological interventions to foster resilience in healthcare professionals. Cochrane Database Syst Rev. 2020;7:CD012527. 32627860 10.1002/14651858.CD012527.pub2 PMC8121081

[32] Salehi AM Khazaei S Masumi M. Reinforcement and maintenance of human resources for health systems during long-term crises: a systematic review of systematic reviews. Emerg Med Int. 2021;2021:9613443. 34754519 10.1155/2021/9613443 PMC8572622

[33] Rhoden DJ Dezordi CCM Husein RAMM. Nurses stress and resilience before and after evaluation for hospital accreditation. Rev Bras Enferm. 2021;75:e20201341. 34852118 10.1590/0034-7167-2020-1341

[34] Radisic G Duncanson E Le Leu R. Improving management of needle distress during the journey to dialysis through psychological education and training-the INJECT study feasibility pilot protocol. Pilot Feasibility Stud. 2022;8:28. 35120560 10.1186/s40814-022-00989-2 PMC8815234

[35] Liang M Chen Q Li Y. Status quo and influencing factors for anxiety, depression, and insomnia among 4 237 nurses in Hunan Province. Zhong Nan Da Xue Xue Bao Yi Xue Ban. 2021;46:822–30. 34565725 10.11817/j.issn.1672-7347.2021.210212 PMC10929976

